# Treatment of life-threatening food allergy

**DOI:** 10.1186/2045-7022-3-S3-O8

**Published:** 2013-07-25

**Authors:** C Nilsson, A Nopp, M Wickman, G Johansson, G Lilja

**Affiliations:** 1 Allergy Department, Sachs’ Children’s Hospital, Stockholm, Sweden; 2 Dept of Clinical Science and Education, Södersjukhuset, Stockholm, Sweden; 3 Dept of Medicine, Clinical Immunology and Allergy, Karolinska Institutet, Stockholm, Sweden; 4 Institute of Environmental Medicine, Karolinska Institutet, Stockholm, Sweden

## Background

Food allergy may be life-threatening and affected individuals have impairment in quality of life. Children with multiple and severe food allergies usually have other allergic manifestations like allergy to furry animals and pollen, causing eczema and asthma. Treatment with the anti-IgE (omalizumab) is indicated for severe allergic asthma. Oral immunotherapy (OIT) has been performed in studies, but has shown to be associated with allergic side-effects. Basophil allergen threshold sensitivity (CD-sens) is suggested to mirror the patient’s allergen sensitivity.

## Methods

Two girls, 16 (N) and 10 (L) years, with repeated life-threatening anaphylaxis from cow’s milk, severe asthma, severe eczema and allergy also to egg, nuts, peanuts, furry animals and pollens, have been treated with omalizumab followed by OIT during tapering down of the omalizumab treatment. The dose of omalizumab was assessed according to the manufacturer recommendations of asthma treatment. CD-sens was performed, before and during the treatment period. The OIT started with 1-5 ml of fresh milk with increase of another 5 ml every week until a normal serving portion of 300 ml was reached.

## Results

CD-sens to milk, high at starting point, was zero three months after omalizumab treatment. A milk challenge was performed and the girls passed the challenge without any symptoms and had 300 ml (L) and 450 ml (N) of cow’s milk, respectively. The girls went through the OIT treatment during protection of omalizumab without any side effects. Milk is now daily consumed without restrictions. After some months with omalizumab both girls experienced an improvement of their asthma, needed lower amount of inhalation steroids, had a normal sleeping pattern and improved concentration at school. L did no longer need extra supported teaching to keep up with schoolwork. Both girls experienced a better quality of their hair and nails.

## Conclusion

Children/teenagers with a “systemic” allergic disease as indicated by multiple allergic manifestations including severe food allergy, asthma, and eczema seem to benefit in general by treatment combining omalizumab and OIT.

## Disclosure of interest

None declared.

